# A Method for Structure–Activity Analysis of Quorum-Sensing Signaling Peptides from Naturally Transformable Streptococci

**DOI:** 10.1007/s12575-009-9009-9

**Published:** 2009-06-11

**Authors:** XiaoLin Tian, Raymond T Syvitski, TianLei Liu, Nadine Livingstone, David L Jakeman, Yung-Hua Li

**Affiliations:** 1 Department of Applied Oral Sciences, Dalhousie University, Halifax, Nova Scotia, Canada; 2 Institute for Marine Biosciences, National Research Council of Canada, Dalhousie University, Halifax, Nova Scotia, Canada; 3 College of Pharmacy, Dalhousie University, Halifax, Nova Scotia, Canada; 4 Department of Microbiology and Immunology, Dalhousie University, 5981 University Ave. Rm5215, Halifax, Nova Scotia, B3H 3J5, Canada

**Keywords:** Quorum sensing, Signaling peptides, Structure–activity analysis, Circular dichroism and nuclear magnetic resonance spectroscopy, *Streptococcus mutans*

## Abstract

Many species of streptococci secrete and use a competence-stimulating peptide (CSP) to initiate quorum sensing for induction of genetic competence, bacteriocin production, and other activities. These signaling molecules are small, unmodified peptides that induce powerful strain-specific activity at nano-molar concentrations. This feature has provided an excellent opportunity to explore their structure–function relationships. However, CSP variants have also been identified in many species, and each specifically activates its cognate receptor. How such minor changes dramatically affect the specificity of these peptides remains unclear. Structure–activity analysis of these peptides may provide clues for understanding the specificity of signaling peptide–receptor interactions. Here, we use the *Streptococcus mutans* CSP as an example to describe methods of analyzing its structure–activity relationship. The methods described here may provide a platform for studying quorum-sensing signaling peptides of other naturally transformable streptococci.

## 1. Introduction

**Figure 1:**
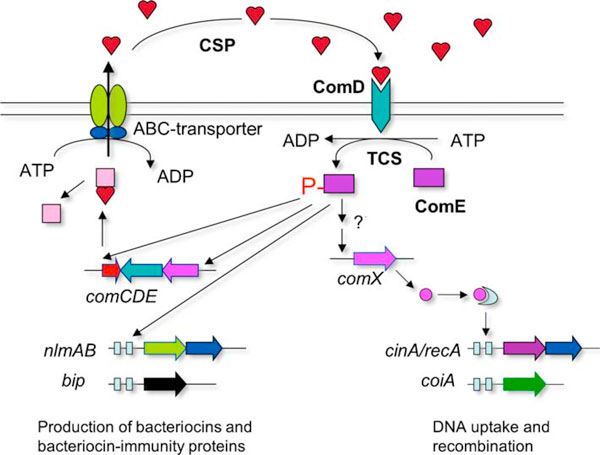
**A schematic diagram describing the quorum-sensing system and its controlled cooperative activities in *Streptococcus mutans***. The signaling peptide or CSP induces quorum-sensing cascade when reaching to a critical concentration. This in turn activates the production of numerous bacteriocins and genetic competence, resulting in killing of other species, DNA release, and gene exchange.

Natural genetic transformation is a process by which bacteria are able to take up and integrate exogenous free DNA from their environment [1,2]. This process enables the recipient organisms to acquire novel genes, thereby promoting the emergence of antibiotic resistance, genetic variation, and rapid evolution of virulence factors [1-4]. Many members of the genus *Streptococcus* are naturally transformable and each depends on a signaling peptide-mediated quorum-sensing system for induction of genetic competence [3-5]. Activation of quorum sensing for genetic competence and other coordinated activities in these species requires at least six gene products encoded by *comCDE*, *comAB*, and *comX* (Figure 1). The *comC* gene encodes a competence-stimulating peptide (CSP) precursor, which is cleaved and exported through a peptide-specific ABC transporter encoded by *comAB*, releasing CSP into a extracellular environment [2-6]. The *comDE* encodes a two-component system consisting of a histidine kinase sensor protein (ComD) and its cognate response regulator (ComE) that specifically senses and responds to CSP. At a critical concentration, the CSP activates autophosphorylation of the ComD of neighboring cells. The phosphate group is then transferred to the ComE, which in turn activates its target genes, including *comX* that encodes a competence-specific sigma factor recognizing a consensus sequence (com-box) at the promoter regions of late competence genes, triggering the signaling cascade for genetic competence [2-5]. The quorum-sensing system in *Streptococcus mutans* represents a unique regulatory mechanism, since this system regulates both bacteriocin production and genetic competence [2,6,7]. In contrast to *S. mutans*, *Streptococcus pneumoniae* requires two separate signaling systems, the ComCDE and BlpRH, to regulate genetic competence and bacteriocin production [8].

Many CSPs from naturally transformable streptococci have been identified and their activity for genetic competence has been validated [2-4]. These signaling molecules are small, unmodified peptides, ranging from 14 to 25 residues in length, and induce powerful strain-specific activity at nano-molar concentrations. Some CSPs have been chemically synthesized and used as a tool to induce genetic competence during molecular cloning. Recently, the structure–activity relationships of CSPs from both *S. pneumoniae* and *S. mutans* have been analyzed [9,10]. The CSPs from these organisms have been found to adopt an amphipathic, α-helical conformation with a defined hydrophobic face that contributes to the CSP specificity. Furthermore, the CSP from *S. mutans* reveals two functional domains [10]. The C-terminal structural motif consisting of a sequence of polar hydrophobic charged residues is crucial for activating the signal transduction pathway, while the core α-helical structure extending from residue 5 through to the end of the peptide is required for receptor binding. Sequence alignment of CSPs from various streptococci shows that almost all CSPs have such a motif at the C-termini [10], suggesting that these CSPs likely have similar functional domains. However, CSP variants have been identified in many species and each induces competence in a highly strain-specific manner. Thus, the CSP variants may provide an excellent model to study peptide–receptor interactions in these bacteria. Here, we extend our technical information and describe methods and protocols for structure–activity analysis of CSP from *S. mutans* by combining activity assays with nuclear magnetic resonance (NMR) and circular dichroism (CD) spectroscopies (*see ***Appendix**). The methods described herein may provide a platform and rationale for analyzing CSPs from other naturally transformable streptococci.

## 2. Materials and Methods

### 2.1. Chemical Synthesis of Signaling Peptides

Efficient structural characterization of signaling peptides by CD and NMR requires purified (≥95% purity) and soluble peptides. In our study, all the peptides were commercially synthesized and purified by reversed-phase high-pressure liquid chromatography. Their identity was confirmed by a mass spectrometry [10]. The peptides were lyophilized and stored at –20°C until use. For CD and NMR studies, each peptide was dissolved in an appropriate solvent. For activity assays, each peptide was freshly dissolved in sterile distilled water at a concentration of 1.0 mM (stock solution), which was further diluted as required.

### 2.2. Sample Preparation

The first step was to optimize sample conditions by adjusting pH, ionic strength, and temperature to mimic physiological conditions with the constraint in which the conditions were compatible with data acquisition [11]. For CD spectroscopy, peptide concentrations were typically on the order of 50 μM with 200 μL of solution [12]. Buffers, co-solvents, and additives (e.g., detergent molecules) were non-chiral, sodium-free, and non-absorbing within the wavelength range (typically 250–180 nm). For NMR studies, buffers, co-solvents, and additives were hydrogen-free or deuterated. Samples could be dissolved in as little as 10 μL, but typically, spectrometers were designed for sample volumes of at least 500 μL. Due to the inherently low sensitivity, concentrations of peptides ranged from 1.0 to 10 mM [13].

In our study, synthetic peptides CSP (UA159sp) and TPC3 for CD and NMR were dissolved in 95/5/0%, 70/0/30%, 30/0/70%, or 0/0/100% aqueous buffer/D_2_O/TFE-*d*_2_, or 95/5 aqueous buffer/D_2_O with 300 mM DPC-*d*_38_ (98 atom %D), where TFE refers to trifluoroethanol and DPC-*d*_38_ refers to perdeuterated dodecylphosphocholine [10,13]. The aqueous buffer used for sample preparation contained 50 mM K_2_HPO_4_/KH_2_PO_4_ at pH 7.0. The concentration of peptides for solutions containing 100% TFE-*d*_2_ or DPC-*d*_38_ was 2 and 5 mM. For diffusion studies, we analyzed the solutions containing peptides and DPC-*d*_38_ by lyophilizing and dissolving them in an equivalent volume of D_2_O (99.9 atom %D) followed by transferring into a 5 mm OD, D_2_O magnetic susceptibility-matched NMR tube (BMS-3; Shigemi, Tokyo). The sample height of 1.2 cm ensured that the entire sample was within the radio frequency coils, which was essential for artifact-free and accurate diffusion measurements.

### 2.3. CD Spectroscopy

CD spectra for peptides and reference solutions were recorded at 298 K on a Jasco J-920 CD spectrometer with a 1-mm quartz cuvette. Spectra were collected and averaged over 16 scans from wavelengths 190 to 250 nm with a 0.1-nm step resolution. CD measurements were performed and analyzed to confirm the effects of TFE on the secondary structure of the peptides [14,15]. In our study, we analyzed the peptides by using CD analysis package DICHROWEB (http://www.cryst.bbk.ac.uk/cdweb/html) [16].

### 2.4. NMR Spectroscopy

Initial experiments in our study included ^1^H-^1^H COSY (correlation spectroscopy) and ^1^H-^1^H TOCSY (total correlation spectroscopy). The COSY spectra were used to correlate ^1^H atoms that were ≤3 bonds apart, and to determine the ^3^J_H_^N^_H_^α^ coupling values between H^N^ and H^α^, whereas the TOCSY spectra were used to correlate all ^1^H atoms within a spin system that were ≤3 bonds apart [17]. In addition, 1D and 2D ^1^H NMR data sets of the peptides were collected on a Bruker AVANCE 500 spectrometer. Unless otherwise stated, ^1^H detected spectra were acquired at a repetition rate of 1.5 s^-1^ at 293 K with water frequency centered on carrier frequency. For structural determination, phase-sensitive 2D ^1^H-^1^H NOESY, ^1^H-^1^H COSY, and ^1^H-^1^H TOCSY data sets (MLEV17) were recorded at 293 K with a presaturation or with a 3–9–19 pulse sequence for water suppression. ^1^H detected 2D DOSY data sets were recorded using a stimulated-echo sequence with bipolar-gradient pulses and 32 t_1_ blocks of 64 transients each. NMR data sets were processed using Bruker XwinNMR 3.5.

### 2.5. Structural Determination

Structural determination of the peptides followed the procedures as described previously [10]. Briefly, spin systems (i.e., amino acid residues) were identified through chemical shifts and TOCSY cross-peak patterns. Distance constraints determined from integration of the 250-ms NOESY spectra were classified into four groups: strong, medium, weak, and very weak corresponding to interproton distance ranges of <2.3, 2.0–3.5, 3.3–5.0, and 4.8–6.0 Å, respectively. The ^3^J_H_^N^_H_^α^ coupling values determined from COSY spectra were averaged over a distribution of dihedral angles. Assessment of hydrogen bonding was carried out on the basis of H^α^_*i*_–H^N^_*i* + 3_, and H^α^_*i*_–H^N^_*i* + 4_ observed NOESY connectivities, dihedral angles, and chemical shift index values. For residues involved in hydrogen bonding, hydrogen bonds between CO_*i*_ and H^N^_*i+4*_ were included as constraints by restraining ***r***_H–O_ to 1.2–1.9 Å and ***r***_N–O_ to 1.8–2.9 Å.

From the NMR-derived structural constraints, two series of structural calculations were performed with or without added hydrogen bonds to assess the effect of adding suspected hydrogen bonds into the structure calculation. All structural calculations were based on previous studies using the XPLOR 3.1 software package [10]. Final calculated structures were energy minimized with 2,000 conjugate gradient steps before processing to the restrained annealing molecular dynamics calculation. A total of 33 and 21 UA159sp and 43 and 36 TPC3 (with and without hydrogen bonds included within the simulation) lowest energy structures were retained that had no violations of NOESY constraints >0.5 Å [18]. The overall quality of these refined structures was examined with the program PROCHECK [19]. Except for random coil sections, all backbone dihedral angles resided in the well-defined, acceptable regions of the Ramachandran plot. Three-dimensional structures of UA159sp and TCP3 determined from NMR were constructed by computer simulation using the VMD software (http://www.ks.uiuc.edu/Development).

### 2.6. Construction of ***comC*** Mutant That Was Defective in Producing CSP

To assay quorum sensing (QS) activation in response to addition of a peptide without interference by endogenous CSP from a wild-type strain, a *comC* mutant that was unable to produce, but still responded to CSP was constructed in *S. mutans* GS5 using the strategy as described previously [20]. Following genetic recombination via allelic exchange, the *comC* deletion mutant was created by insertion of a spectinomycin (Spec^r^) resistance cassette [21] into the *comC* gene of strain GS5. The mutant was then confirmed by polymerase chain reaction (PCR) and sequencing of the junction site of the Spec cassette as described previously [20]. The resulting mutant was named SMdC and was used as a negative background strain of signaling peptide (CSP) for CSP-dependent assays of genetic competence, *lacZ* reporter, and bacteriocin production.

### 2.7. Construction of ***lacZ*** Transcriptional Reporter Strains

To assay QS activation in response to addition of a peptide, we constructed two types of *lacZ* transcriptional reporter strains that represented two levels of QS-activated gene expression. The first was a *lacZ* reporter gene fused to the *comDE* promoter, which allowed monitoring of the activity of the *comDE* promoter in response to a test peptide. The second strain was a *lacZ* reporter gene fused to the promoter of *nlmAB*, the gene encoding a nonlantibiotic bacteriocin and its expression directly controlled by the ComE [6,22]. This strain allowed assay of *comDE*-controlled bacteriocin production.

For the first reporter strain, we simply transformed previously constructed vector pYH2 [10] into the *comC* mutant (SMdC) to generate SMdC-pYH2 (P*comDE::lacZ*, *comC*^-^, Spec^r^, Kan^r^). pYH2 was constructed in such a way that the *comDE* promoter was fused to a promoterless *lacZ* gene on the backbone of *Streptococcus–Escherichia coli* shuttle vector pSL. The vector pSL was also transformed into the same mutant SMdC and used as a background control (SMdC-pSL). These strains were then assessed for *lacZ* reporter activity in response to addition of peptides.

For the second reporter strain, we generated a chromosomal nlmAB–lacZ fusion strain by transforming a P*nlmAB::lacZ* fusion construct pOMZ47 [6] into both *comC* mutant SMdC and wild-type GS5. The resulting reporter strains were confirmed by PCR and sequencing of the fusion sites as described previously [6]. The confirmed reporter strains were respectively named SMdC-PnlmAB (P*nlmAB::lacZ*, *comC*^-^, Kan^r^, Spec^r^) and SMGS5-PnlmAB (P*nlmAB::lacZ*, Kan^r^). The strain SMGS5-PnlmAB was used a positive control.

### 2.8. CSP-Dependent Transformation Assay

To determine if synthetic peptides activated quorum sensing for induction of genetic competence, we used the *comC* deletion mutant that was unable to produce, but still responded to CSP, to assay peptide-dependent genetic transformation using the method as described previously [2,10]. A streptococcal suicide vector pVA-gtfA, which harbored a 2.4-kb fragment of the *S. mutans gtfA* gene and an erythromycin resistance marker, was used as transforming DNA. The wild-type GS5 was used as a positive control, while both ComC (SMdC) and ComE mutants (SMdE) were used as negative controls. The wild-type strain was grown on Todd–Hewitt yeast extract (THYE) medium, whereas the mutants were maintained on THYE plus spectinomycin. When culture reached to early mid-log phase (OD_600_ ≈ 0.25), an aliquot of CSP was added into the culture at a final concentration of 50 nM (AC_50_ ≈ 0.25 nM). After 20 min of incubation at 37°C, an aliquot of transforming DNA of plasmid pVA-gtfA conferring erythromycin resistance was added at a final concentration of 1 μg mL^-1^. The culture was incubated for additional 2 h before plated on THYE agar plates plus 10 μg mL^-1^ erythromycin. Prior to addition of transforming DNA, an aliquot of the cell suspension was plated on THYE plates containing the same antibiotic to monitor spontaneous mutation. Transformation frequency was expressed as percentage of transformants against total viable recipient cells per milliliter cell suspension.

### 2.9. β-Galactosidase Activity Assay

A *lacZ* reporter assay was performed to monitor quorum-sensing activation using the same method as described previously [10]. The newly constructed *lacZ* reporter strains were used to assay and quantify β-galactosidase activity in response to test peptides. Aliquots of samples were taken to prepare cell lysates at different time points *T*_0_, *T*_15_, *T*_30_, *T*_60_, and *T*_120_ after addition of a test peptide. Protein concentrations of the supernatants were determined by Bio-Rad protein assay (Bio-Rad). Specific β-gal activities (*A*_420_ min^-1^ mL^-1^ mg^-1^ protein) were calculated from triplicate samples from two independent experiments. The data from the β-gal activity experiments were then normalized against negative or background controls and plotted as percentages of maximal activation versus log peptide concentrations. The half maximal activation concentration (AC_50_) was determined from the sigmoidal dose–response curve using Prism 4 (Graphpad, San Diego, CA, USA).

### 2.10. Assay for QS-Controlled Bacteriocin Production

The *S. mutans comC* mutant SMdC as well as its parent GS5 and wild-type strain UA159 (positive controls) were used to assay for bacteriocin production using a modified agar plate method [7,23-25]. Briefly, an aliquot of mid-log phase cells of the *S. mutans* wild-type strains and mutant SMdC grown under a condition with or without use of CSP or a test peptide were stabbed onto THYE agar plates. The plates were incubated anaerobically at 37°C for 6 h before carefully overlaid with 100 μL of cell suspension (about 10^6^ CFU/mL^-1^) of an indicator strain *Streptococcus sanguinis* SK108. The plates were further incubated anaerobically at 37°C for 24 h before inspection of bacteriocin production. The *S. mutans* strains showing an inhibitory zone around the stabs were scored as positive for bacteriocin production. The plates were then photographed for records.

## 3. Results and Discussion

### 3.1. CD and NMR Structural Analyses of the Signaling Peptides

CD and NMR spectroscopy are non-destructive and non-invasive techniques that have been widely used to determine secondary folding features of proteins or peptides [14-17]. In addition, NMR spectroscopy records data from "spin-active" nuclei, such as ^1^H, ^13^C, ^15^N, or ^31^P. These nuclei are sensitive to their chemical environment, which is determined by the type and number of bonds and the position and proximity of other chemical entities and the solvent. Thus, NMR is a powerful tool that can be used to determine atomic level structural information, dynamics (chemical exchange, motional fluctuations, proton exchange), ligand binding, multimeric states, and effects of competitive receptors [11,15,26].

**Figure 2:**
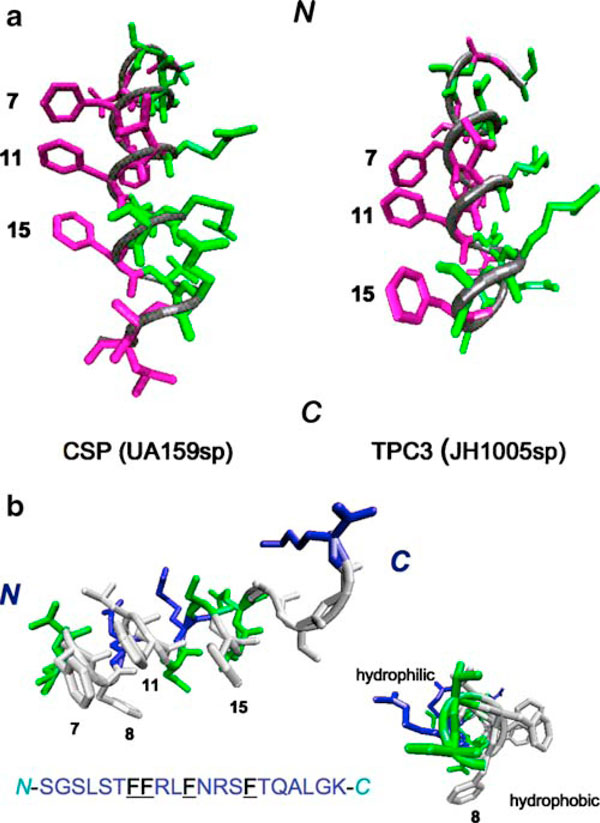
**Computer-simulated three-dimensional structures of UA159sp and TPC3 determined by NMR and CD analyses**. Only the α-helical portions of the peptides with designated hydrophobic and hydrophilic residues are presented. **a** A comparison in three-dimensional structures between UA159sp (CSP) and TPC3 that lacked the proposed C-terminal motif. **b** UA159sp viewed from different angles indicates the positions of phenylalanine (F) residues on the core of α-helix and C-terminal motif of slightly motional freedom.

Using CD and NMR spectroscopy, we have determined the three-dimensional structures of two signaling peptides, UA159sp from *S. mutans* UA159 and a C-terminally truncated peptide TPC3 from JH1005 defective in genetic competence [10]. Initial NMR data for both peptides were recorded in aqueous buffer to provide an assessment of the structural nature of the peptides. In the presence of water, both peptides were not soluble enough for extensive NMR studies. In TFE, however, these peptides formed well-defined α-helices from residues Leu4-Gly20 of UA159sp and Ser5-Thr16 of TPC3 (Figure 2). Both UA159sp and TPC3 formed a similar structure in TFE and DPC-*d*_38_ micelles, although the C-terminal three residues of TCP3 were truncated. The structural coordinates for two peptides have been deposited in the RCSB Protein Data Bank (http://www.pdb.org/) with RCSB ID code rcsb039055 or PDB ID code 2I2J for UA159sp and RCSB ID code rcsb039053 or PDB ID code 212H for TPC3.

An important feature observed from structural analyses was that both UA159sp and TPC3 formed amphipathic α-helices with a well-defined hydrophobic face characterized by a row of four highly hydrophobic phenylalanines (F7, F8, F11, and F15) on one side and hydrophilic residues on another side (Figure 2). The hydrophobic face comprised about 40% of the surface of the helix, whereas hydrophilic and charged residues comprised the remainder of the surface. A major structural difference between UA159sp and TPC3 was that UA159sp showed a clear C-terminal motif of slightly more motional freedom than TPC3. From structures of other similar binding peptides, a well-defined row of hydrophobic residues on an amphipathic α-helix is common and necessary for ligand binding to the receptor protein [12,19]. Our work suggests that the quorum-sensing signaling peptides from *S. mutans*, probably from other naturally transformable streptococci, fall into the FXXFF motif family, a general protein–protein interaction motif, where the interaction is made through a hydrophobic face formed by the hydrophobic residues packed into a hydrophobic pocket of the receptor [11,12,26]. The fact that the amphipathic helical structure is reflected in the binding and induction of quorum-sensing suggests that the peptide may be helical within the active site of the receptor. The membrane environment may help to form the helical structure, facilitating binding to the receptor. We have chosen to dissolve the peptides in widely used solvent TFE because it improves structural stability of peptides and is considered to be biologically relevant [27]. TFE is also thought to mimic a protein receptor environment by stabilizing helices in regions with intrinsic α-helical propensity that are likely to form helices when binding to their protein partner.

### 3.2. Signaling Peptide–Receptor Activation in Newly Constructed Strains

**Figure 3:**
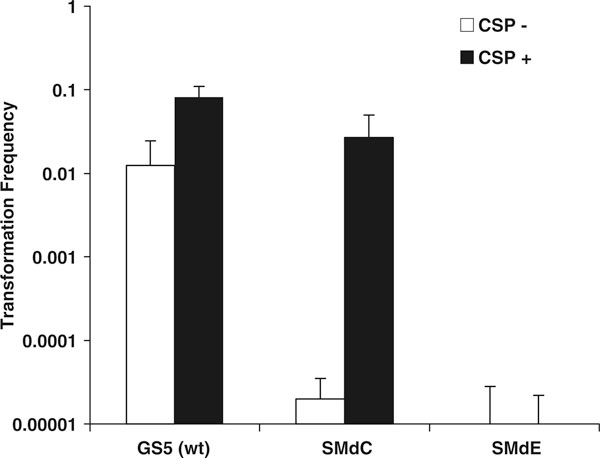
**Induction of genetic competence of the *comC* mutant (*SMdC*) in response to addition of CSP**. *S. mutans* wild-type GS5 was used as a positive control, while the *comE* deletion mutant (*SMdE*) was used as a negative control.

**Figure 4:**
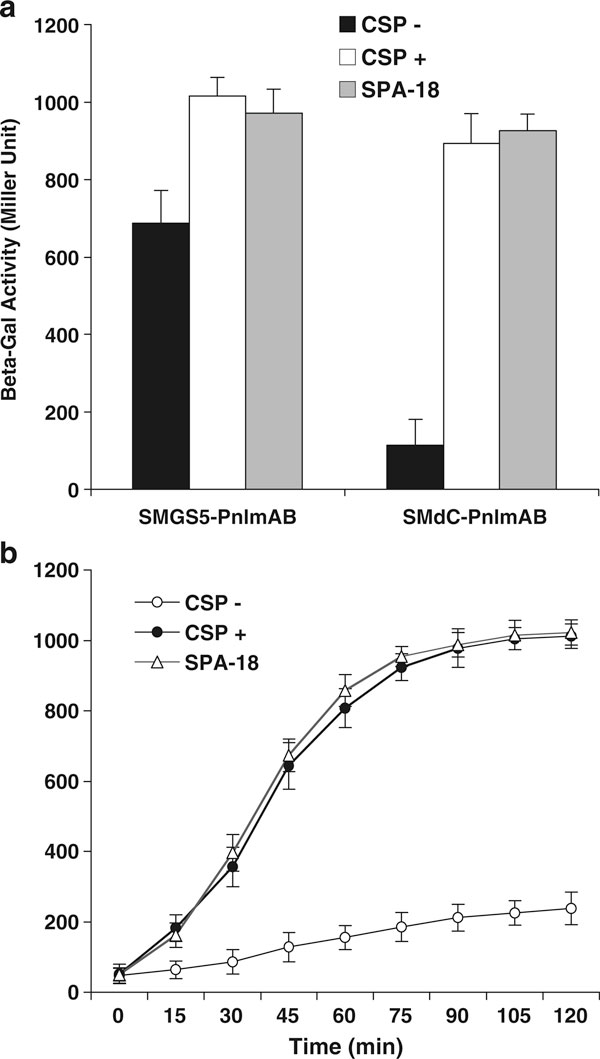
**Constructed *lacZ* reporter strains, SMdC-PnlmAB and SMGS5-PnlmAB, express predicted β-gal activity in response to CSP or agonist SPA-18**. **a** The expression of β-gal activity in SMdC-PnlmAB is CSP-dependent, which is in contrast to strain SMGS5-PnlmAB. **b** A time-course experiment indicates that SMdC-PnlmAB expresses the highest levels of β-gal activity around 90 min after the addition of CSP or SPA-18 and maintains such high levels of the activity for at least 2 h.

In previous studies, we exclusively used *S. mutans* UA159 and its derivatives to assay QS activation in response to signaling peptides [2,10]. However, this genome sequence reference strain is not a typical producer for CSP-induced bacteriocins [7,24,25]. To construct a *comC* mutant that allowed us to assay CSP-dependent bacteoricin production, we transformed the previously generated *comC* deletion construct [20] into *S. mutans* GS5, a wild-type strain that was demonstrated to produce all known QS-controlled bacteriocins [6,7,22]. The new mutant, named SMdC, was confirmed to have the internal region of *comC* replaced by a spectinomycin (Spec^r^) resistance cassette. The mutant was confirmed to be defective in quorum sensing for induction of genetic competence (Figure 3) and bacteriocin production (Figure 4), unless CSP or a peptide agonist was added into the culture. Our work confirmed that this mutant provided an excellent negative background, enabling the assay of peptide-dependent QS activation without interference by endogenous CSP.

We also used this mutant (SMdC) as a host to generate two types of *lacZ* transcriptional reporter fusion strains to assay the promoter activity of QS-controlled target genes, *comDE* and *nlmAB*, in response to addition of CSP. The *lacZ* reporter strain, SMdC-pYH2 (P*comDE::lacZ*, *comC*^-^, Spec^r^, Kan^r^), along with its background control strain SMdC-pSL (*comC*^-^, Spec^r^, Kan^r^) were then assayed for β-galactosidase activity. The results confirmed that upon addition of CSP strain SMdC-pYH2 was induced to express predicted β-gal activity at similar levels to those of the previously constructed *lacZ* reporter strain in UA159 [10]. In contrast, the background control strain SMdC-pSL did not show significant difference in the expression of β-gal activity. To assay QS activation of the downstream genes controlled by the ComCDE signaling transduction pathway, we also constructed two *nlmAB*–*lacZ* fusion strains by transforming a previously generated fusion construct P*nlmAB::lacZ *[6] into SMdC-GS5 and parent GS5. These strains were genetically confirmed and named SMdC-PnlmAB (P*nlmAB::lacZ*, *comC*^-^, Kan^r^, Spec^r^) and SMGS5-PnlmAB (P*nlmAB::lacZ*, Kan^r^), respectively. The β-gal activity assay showed that prior to addition of CSP SMdC-PnlmAB was defective in expressing a normal level of β-gal activity (Figure 4a). Upon addition of CSP, however, SMdC-PnlmAB was rapidly induced to express an increasing level of β-gal activity and reached to the highest level around 90 min after addition of CSP (Figure 4b). Interestingly, SMdC-PnlmAB still maintained such a high level of the activity for at least 2 h. Clearly, such expression of β-gal activity appeared to be a major difference between this reporter strain and SMdC-pYH2, in which the expression of β-gal activity declined around 40 min after the addition of CSP [10]. However, the expression of β-gal activity by SMdC-PnlmAB was highly inconsistent with those of other reports [6,7], suggesting that the expression of *nlmAB* gene was likely enhanced by an unknown factor following initial activation by CSP. Thus, both *lacZ* reporter strains representing two levels of gene regulation provided us with a sensitive detecting system to assay and identify signaling peptide agonists.

### 3.3. Application of the Methods to Identify Signaling Peptide Agonists

**Figure 5:**
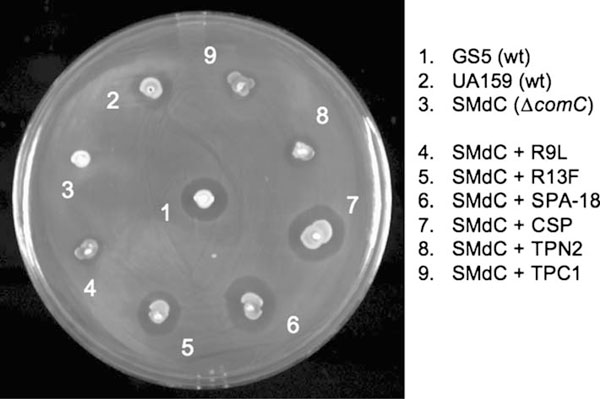
**QS-controlled bacteriocin production in *S. mutans* strains**. Only peptides CSP, SPA-18 (*5*), and R13F (*4*) induce the same level of bacteriocins as GS5 (*1*).

Based on the three-dimensional structures of *S. mutans* wild-type signaling peptide UA159sp (CSP) and C-terminally truncated peptide TPC3 from mutant JH1005 defective in genetic competence, we designed and synthesized a series of truncated peptides and peptides with amino acid substitutions [10]. By functional analysis of these peptides, we found that CSP from *S. mutans* displayed two functional domains. The C-terminal structural motif consisting of a sequence of polar hydrophobic charged residues is crucial for activating the signal transduction pathway, while the core α-helical structure extending from residue 5 through the end of the CSP is required for receptor binding. The AC_50_ of CSP (UA159sp) was determined to be about 25 nM [10]. With these data, we developed a rationale to design and synthesize several new peptide agonists (Table 1). By assaying their activity in quorum-sensing activation using the methods described here, we found that hydrophobic residues, leucine (L) or phenylalanine (F), could replace the hydrophilic residues at positions 9, 12, or 13 of the core α-helix of CSP without significant change of its activity (Table 1). In contrast, peptides, F7Q, F11Q, and F15Q that had hydrophobic phenylalanines at positions 7, 11, or 15 replaced by a hydrophilic residue, glutamine (G), failed to activate quorum sensing [10]. In addition, we found that alanine (A) at position 18 appeared to be important for the activity, since a deletion or replacement of this residue abolished the peptide activity. The evidence strongly suggests that the maintenance of the hydrophobic face of the signaling peptide is crucial for receptor recognition. These results are consistent with the results of Havarstein and colleagues [9], who have found that the hydrophobic patch of CSP1 and CSP2 of *S. pneumoniae* mainly contributes to the receptor recognition and the specificity. Interestingly, the hydrophobic patch in the *S. pneumoniae* CSP1 also consists of three highly hydrophobic phenylalanines at positions 7, 8, and 11 and an isoleucine at position 12. In contrast to *S. pneumoniae*, however, CSP receptor recognition in *S. mutans* appears to be less specific among strains. For example, the peptides with a truncation of N-terminal residues, such as TPN1 or TPN2, or C-terminal residue TPC1, also functioned as agonists with AC_50_ values similar to CSP [10]. These appear to be consistent with the report of Allan et al. [28], who have found no strict correlation between the CSP genotype and the ability to induce quorum sensing among *S. mutans* strains. Together, these findings suggest that the *S. mutans* CSP and its variants may have less recognition specificity of interacting with its receptor protein. Unlike *S. pneumoniae* that requires two independent signaling systems, the ComCDE and BlpRH, to regulate genetic competence and bacteriocin production, *S. mutans* only uses one signaling system with more flexible peptide–receptor interaction to regulate these phenotypes. We speculate that more peptide variants may be found in *S. mutans* isolates, and they likely function in the same fashion as CSP (UA159sp) to activate quorum sensing for bacteriocin production and genetic competence. To support this speculation, we designed a shorter peptide agonist, SPA-18, to assay its activity. We found that this shorter peptide could completely replace CSP (UA159sp) in activating quorum sensing (Figure 4), genetic competence (data not shown), and bacteriocin production (Figure 5).

**Table 1:** Peptide analogs and their agonist activity in quorum sensing

Name of peptide	**Amino acid sequences**^**a**^	**AC**_**50 **_**(nM)**^**b**^
CSP (UA159sp)	SGSLSTFFRLFNRSFTQALGK	25
TPC3 (JH1005sp)	SGTLSTFFRLFNRSFTQA- - -	268
Peptide agonists		
R9L	SGSLSTFF**L**LFNRSFTQALGK	32
N12L	SGSLSTFFRLF**L**RSFTQALGK	30
R13F	SGSLSTFFRLFN**F**SFTQALGK	26
TPC1	SGSLSTFFRLFNRSFTQALG –	29
TPN2	- - SLSTFFRLFNRSFTQALGK	32
SPA-18	- - SLSTFFRLFN**F**SFTQALG -	25

^a^Modifications of amino acid residues are in bold and underlined

^b^The AC_50_ values were calculated from a dose–response curve using Graphpad Prism 4

## 4. Conclusions

Quorum-sensing signaling systems involving the interaction between a signaling peptide and its cognate histidine kinase receptor are widely distributed in Gram-positive bacteria. Structure–activity analysis of signaling peptides and their analogs may provide clues for understanding the specificity of peptide–receptor interactions in these bacteria. In this report, we have used the *S. mutans* CSP as an example to describe the methods for structure–activity analysis of quorum-sensing signaling peptides by combining activity assays with CD and NMR spectroscopy. By analyzing these peptides, we have identified several signaling peptide agonists that activate quorum-sensing cascade for induction of bacteriocin production and genetic competence in *S. mutans*. The methods described herein may provide a platform for analyzing structure–activity relationships of signaling peptides from many other naturally transformable streptococci.

## Abbreviations

CSP: Competence-stimulating peptide; QS: Quorum sensing; CD: Circular dichroism; NMR: Nuclear magnetic resonance; HPLC: High-pressure liquid chromatography; TFE: Trifluoroethanol; DPC: Dodecylphosphocholine; COSY: Correlation spectroscopy; TOCSY: Total correlation spectroscopy; NOESY: Nuclear Overhauser effect spectroscopy; DOSY: Diffusion-ordered spectroscopy; SDS: Sodium dodecyl sulfate; ONPG: *O*-Nitrophenyl-D-galactopyranoside

## Appendix

### Protocol 1. Structural Determination of CSPs by CD Spectroscopy

1. Prepare 2–5 mL of a stock solution (A) to the appropriate concentrations with non-chiral, sodium-free buffers, solvents, and additives.

2. Dissolve lyophilized peptide in 1 mL of stock solution A at a concentration of 500 μM (stock solution B). For structural determination, the stock solutions A and B must have identical concentrations of buffers, solvents, and additives.

3. Dilute stock solution B to make the peptide concentration of 100 μM. Note: The quality of a CD spectrum is dependent on the peptide concentration. Low peptide concentrations result in poor S/N, whereas high concentrations saturate the detector leading to artifacts.

4. Place the peptide solution in a cuvette and ensure the beam path of the cuvette is clean. Record and average the CD spectra in the range of 180 to 250 nm until a smooth CD spectrum is obtained (e.g., 16 scans with a 1.0-nm increment).

5. Further dilute the peptide solution with A to 50 μM, and record the CD spectra in a similar manner. Adjust the peptide concentration and recording CD spectra so that the receiver is not saturated, but there is enough signal for a spectrum.

6. Record the CD spectrum of solution A using identical spectrometer conditions.

7. Subtract the CD spectra of solution A from that obtained for the peptide solution by using CD software.

8. Analyze the CD spectra to determine the secondary structural features. There are several CD analysis programs and web-based analysis interfaces such as CD analysis package DICHROWEB (http://www.cryst.bbk.ac.uk/cdweb/html). Typically, the software is capable of multiple analysis algorithms.

### Protocol 2. Structural Determination of CSPs by ^1^H NMR Spectroscopy

1. Dissolve purified peptides to a concentration of >1 mM in appropriate deuterated buffers, solvents, and additives.

2. Record ^1^H-^1^H COSY, TOCSY, NOESY, DOSY spectra with appropriate parameters.

3. Typically, NMR spectra are processed using sin-bell squared apodization functions to "sharpen" the signals. Process the spectra with the appropriate functions and phase corrections to produce a defined spectrum.

4. Identify as many individual amino acids as possible by correlating protons though bonds using COSY and TOCSY spectra.

5. Determine sequence-specific assignments by connecting H^N^_*i*_ to H^α^_*i -* 1_ and/or H^N^_*i*_ to H^N^_*i* ± 1_ protons with the NOESY spectra. Determine longer range, non-sequential ^1^H-^1^H correlations from NOESY spectra.

6. Once a number of sequence specific residues have been assigned, integrate the appropriate NOESY cross-peaks and estimate the corresponding distance.

7. If possible, determine the ^3^J_H_^N^_H_^α^ coupling values from the COSY spectra. It may be necessary to fit the line shape to determine an accurate coupling value. With the ^3^J_H_^N^_H_^α^ coupling values, estimate the dihedral angles (if ^3^J_H_^N^_H_^α^ coupling value is <6.0 Hz, α-helix; ^3^J_H_^N^_H_^α^ values between 6.0 and 8.0 Hz, random coil; and ^3^J_H_^N^_H_^α^ values >8.0 Hz, β-sheet structures).

8. Using a simulated annealing molecular dynamics program, determine a preliminary family of non-violating structures from determined distances and dihedral angles.

9. Based on the preliminary structure, examine the NOESY spectrum and assign any ambiguous peaks. Then, recalculate structures in an iterative fashion.

### Protocol 3. Signaling Peptide-Dependent Competence Assay

1. Inoculate a single colony of SMdC mutant into 2 mL broth of Todd–Hewitt yeast extract (THYE) (BBL^®^; Becton Dickinson, MD, USA) supplemented with 500 μg/mL of spectinomycin. The culture is incubated at 37°C overnight.

2. Transfer the overnight culture into 2 mL of pre-warmed, fresh THYE broth in 1:20 dilution. Each culture is incubated for 2–3 h to reach to the early mid-log phase (OD_600_ ≈ 0.2–0.3).

3. The culture is divided into two. One is added with a synthetic peptide at a final concentration of 50 nM, while another (negative control) is added with an equal volume of distilled water.

4. The cultures are incubated at 37°C for 15 min before added with a transforming DNA (plasmid pVA-gtfA) at the final concentration of 1 μg/mL. The cultures are incubated at 37°C for additional 2 h.

5. A 100-μL cell suspension from each culture is spread on a THYE agar plate plus erythromycin (10 μg/mL) after gentle vortexing.

6. An aliquot of the cell suspension is taken, diluted, and inoculated on THYE agar plates with no antibiotic to determine total viable cell counts.

7. All the plates are incubated at 37°C for 2 days before assessment of transformation frequency, which is expressed as percentages of transformants (erythromycin-resistant colonies) against total recipient cells per millimeter cell suspension.

8. An aliquot of the cell suspension prior to addition of the transforming DNA is also taken and inoculated on THYE plates supplemented with the same antibiotic to determine spontaneous mutation.

### Protocol 4. ***lacZ*** Reporter Assay for Signaling Peptide–Receptor Activation

1. Inoculate a single colony of each of the constructed *lacZ* transcriptional reporter strains into 2 mL THYE broth for overnight culture.

2. Transfer the overnight culture into 40 mL of pre-warmed, fresh THYE broth in 1:20 dilution. Each culture is incubated for 2–3 h to reach the early mid-log phase (OD_600_ ≈ 0.2–0.3).

3. The culture is then divided into two. One is added with a synthetic peptide at a final concentration of 50 nM, while another (negative control) is added with an equal volume of distilled water.

4. Aliquots of samples are taken from each culture at time points of *T*_0_, *T*_15_, *T*_30_, *T*_60_, and *T*_120_ following the addition of a test peptide.

5. The cell suspensions are immediately centrifuged at 10,000×*g* at 4°C for 10 min and the pellet is re-suspended into chilled 50 mM Tris–HCl buffer (pH 7.5) containing 0.27% (*v*/*v*) β-mercaptoethanol in a total volume of 1 mL in a Fastprep tube.

6. Cells are permeabilized by a Fastprep instrument at a setting of 6.0 for 30 s after addition of 50 μL chloroform and 20 μL of 0.1% sodium dodecyl sulfate (SDS). The samples are immediately centrifuged at 10,000×*g* at 4°C for 5 min.

7. An aliquot (100 μL) of the supernatant from each sample is added in triplicate onto a 96-well microtiter plate. Then, 50 μL of *O*-nitrophenyl-D-galactopyranoside is added into each well at a final concentration of 80 μM.

8. The reactions are incubated for 1 h, stopped by adding 50 μL of 1 M Na_2_CO_3_ into each well, and then quantified by a multi-detection micro-plate reader (Synergy) at 420 nm. The reading results are saved for calculation of specific β-gal activity.

9. An aliquot (100 μL) of the supernatants is also transferred into a microtiter plate for determination of the concentration of proteins using Bio-Rad protein assay (Bio-Rad).

10. Specific β-gal activity of each sample is calculated from triplicate samples from two independent experiments and the data are then normalized against negative or background controls. Specific β-gal activity is expressed as Miller units (*A*_420_ per min^-1^ mL^-1^ mg^-1^ protein).

11. The data from specific β-gal activity are then plotted as percentages of maximal activation versus log peptide concentrations. The half maximal activation concentration (AC_50_) is determined from Sigmoidal dose–response curves using Prism 4 (Graphpad).
